# Unacylated ghrelin prevents mitochondrial dysfunction in a model of ischemia/reperfusion liver injury

**DOI:** 10.1038/cddiscovery.2017.77

**Published:** 2017-12-04

**Authors:** Alberto Rossetti, Gabriele Togliatto, Anabela P Rolo, João S Teodoro, Riccarda Granata, Ezio Ghigo, Amedeo Columbano, Carlos M Palmeira, Maria Felice Brizzi

**Affiliations:** 1 Department of Medical Sciences, University of Turin, Turin, Italy; 2 Department of Life Sciences and Center for Neurosciences and Cell Biology, University of Coimbra, Coimbra, Portugal; 3 Department of Biomedical Sciences, University of Cagliari, 09124 Cagliari, Italy

## Abstract

Ischemia/reperfusion (I/R) injury is a common cause of liver dysfunction during hepatectomy, liver transplantation procedures and in generalized shock. Although effort has been dedicated to rescuing tissue damage in these clinical settings, there is still an urgent need for an effective treatment to protect the liver from the burden of I/R injury. In this study, we have investigated the potential clinical impact of unacylated-ghrelin (UnAG) in a liver I/R rat model. Particular attention has been paid to mitochondria. We demonstrate that UnAG was able to reduce the lag-phase time in response to ADP administration and increase oxygen consumption in *ex vivo* experiments using liver mitochondria recovered from rats subjected to I/R. Moreover, we found that UnAG rescued the expression of a key regulator of mitochondrial morphology and electron transport chain function; the optic atrophy 1 (Opa1) protein. Cytochrome *c* oxidase (COX), ATP synthase (complex V) activity and mitochondrial permeability transition pore (mPTP) opening were also affected by UnAG administration *in vivo*. An *in vitro*, hepatic I/R model was used to validate these data. We demonstrate that UnAG upregulates the expression of Cox subunit IV (CoxIV) and increases cellular ATP content. This results in Bcl-2 upregulation and protection against apoptosis. Opa1 silencing shows that Opa1 is crucial for a UnAG-induced increase in cellular ATP content, apoptosis resistance, Bcl-2 and CoxIV expression. Finally, we show that UnAG improves Opa1’s interaction with MIC60 in the I/R setting, hinting at its role in cristae shape regulation. Our results demonstrate that UnAG administration rescues the intrinsic mitochondrial pathway triggered by I/R damage. Opa1’s contribution in mediating this effect is also reported. This suggests that UnAG can interfere with mitochondrial dysfunction, via Opa1, in a preclinical liver I/R model. We therefore provide the rationale for exploiting UnAG as an alternative means to rescuing mitochondrial damage and organ dysfunction.

## Introduction

Ischemic organ damage is a leading cause of morbidity and mortality worldwide.^1,2^ Hepatic ischemia-reperfusion (I/R) injury occurs in several clinical settings, including liver transplantation, liver resection and systemic shock.^3,4^ Although liver transplantation is one of the most efficient treatments for end-stage hepatic disease, up to 30% of primary graft dysfunction in this specific clinical setting has been attributed to I/R injury.^3^ The term I/R injury has been introduced to describe the initial tissue damage caused by a deprivation of blood flow and oxygen and the subsequent severe inflammatory responses in the early phase of organ reperfusion. It is well established that mitochondria are the central target of I/R injury.^5^ Mitochondria are powerhouse organelles which are involved in the oxidative phosphorylation (OXPHOS) process via the electron transport chain (ETC). This translates into the energy-yielding metabolism of aerobic organisms.^6^ The ETC drives and couples electrons flow to protons pumping into the mitochondrial inter-membrane space, which results in chemiosmotic gradient (Δ*ψ*) generation and furnishes the free energy for ATP synthesis. ATP synthase, a membrane protein complex that is also called ETC complex V, is the machinery which phosphorylates ADP by bringing back protons. The ETC is made up of four major respiratory complexes, besides complex V. One of these, Cytochrome *c* oxidase (COX or *complex IV*) is considered a key regulator of the whole OXPHOS, since it acts as a final effector of the entire respiratory chain.^7^ In physiological conditions, functional mitochondria require a balance between fission and fusion as well as proper mitochondrial cristae density. Optic atrophy 1 (Opa1), a profusion dynamin-related protein of the inner mitochondrial membrane (IM), is one of the most important mediators of mitochondrial homeostasis.^8^ Data from Opa1 genetic silencing demonstrated its crucial role in cristae organization and mitochondrial respiratory efficiency.^9,10^

A multi-subunit mitochondrial contact site and cristae organizing system (MICOS) complex has recently been described at cristae junctions (CJ).^11–13^ MICOS has been shown to play a crucial role in forming and maintaining the architecture of the mitochondrial IM. The IM is an energy-coupling membrane and the central site for cellular ATP production.^14–16^ The formation of multimeric complexes, formed by Opa1 and MIC60, has more recently been described as a crucial regulator of crista shape.^17^

It has been well documented that a lack of oxygen availability during I/R damage results in ETC dysfunction and eventually in the early exit of electrons and protons which, in turn, translates into ROS production and diminished mitochondrial coupling efficiency.^18^ Contextually, mitochondrial proteases activate and degrade Opa1, which initiates a vicious cycle, including mitochondrial fission and fragmentation, that further leads to ATP depletion, cell swelling and necrotic cell death.^19^ Owing to the crucial role that mitochondria play in mediating tissue damage in the I/R setting, a great deal of effort has been dedicated to identifying novel therapeutic options to protect mitochondria and avoid I/R-associated adverse effects and organ failure. However, none have succeeded.^20,21^

Unacylated-ghrelin (UnAG) is a form of ghrelin and is mainly produced in the stomach.^22^ UnAG differs from acylated ghrelin (AG) in terms of its biological activity and its molecular receptor has yet to be characterized. UnAG is the more abundant circulating form of ghrelin, which exerts anti-oxidant effects on diabetic-derived endothelial progenitor cells.^23^ Moreover, UnAG was also found to protect muscle and endothelial cells in I/R preclinical models by regulating superoxide dismutase 2, a key mitochondrial anti-oxidant enzyme.^24,25^ Evidence for the protective role that ghrelin plays against liver I/R damage has been provided.^3^ Based on these considerations, the aim of the present study is to investigate the impact of naturally occurring UnAG in protecting the liver from I/R injury. Particular attention has been paid to analyzing UnAG action on mitochondrial functions.

## Results

### UnAG affects mitochondrial membrane potential (Δ*ψ*) and respiration

In a physiological context, ETC activity eventually results in the generation of an electrochemical gradient (Δ*ψ*) between the mitochondrial matrix and the intermembrane space. Δ*ψ* drives protons back to the matrix and this process is coupled with ATP synthesis.^26^ The beneficial vascular and muscle effects reported in ischemic settings in response to UnAG treatment,^24,25^ led us to investigate its therapeutic potential in Wistar rat livers exposed to I/R.

**Figure 1:**
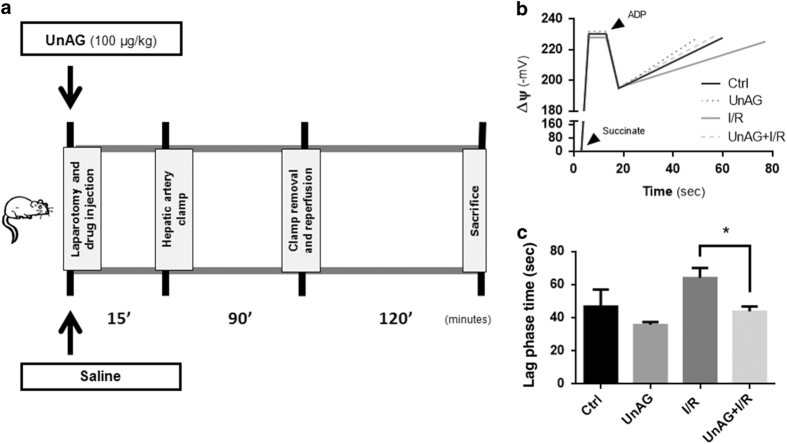
Evaluation of liver mitochondrial membrane potentials (Δ*ψ*) and lag phase time. (**a**) Hepatic I/R injury experimental design: 10 Wistar rats underwent vascular clamp occlusion of the artery (ischemia), perfusing 70% of the liver. After 90 min of ischemia, the clamp was removed and reperfusion allowed for 2 h. Animals were then killed and mitochondria were isolated from the 70% of liver exposed to I/R and from the remaining 30%, which was used as an internal control. Either UnAG (100 *μ*g/kg) or saline were injected into the hepatic artery 15 min before ischemia. (**b**) TPP^+^ electrode traces of liver mitochondria recovered from animals subjected to I/R or not, either treated or untreated with UnAG (100 *μ*g/kg): at time 0 the mitochondrial preparation was energized with succinate, and Δ*ψ* quickly reached a physiological value. The time required for ETC to reestablish a pre-phosphorilative Δ*ψ* value after ADP administration corresponds to the Lag Phase time. Data are reported as mean±S.E.M. and representative of five different experiments, performed in triplicate (*N*=5). (**c**) Quantitative analysis of the Lag Phase time values, evaluated in liver mitochondria recovered from animals treated as indicated (*P<0.05 UnAG+I/R *versus* I/R). Data are reported as mean±S.E.M. and representative of five different experiments, performed in triplicate (*N*=5).

To this end, rats were injected with UnAG 15 min before I/R induction. After 90 min, the clamp was removed and reperfusion allowed for 2 h (Figure 1a). Mitochondria were collected and analyzed for different functional parameters. As shown in Figures 1b and c, while no significant alterations were observed regarding mitochondrial membrane potential and ADP-induced depolarization (Figure 1b), UnAG was able to reduce the lag phase time in response to ADP administration (Figure 1c). These effects are independent of UnAG binding to mitochondria as no significant alterations were registered upon the incubation of isolated mitochondria with UnAG (data not shown).

**Figure 2:**
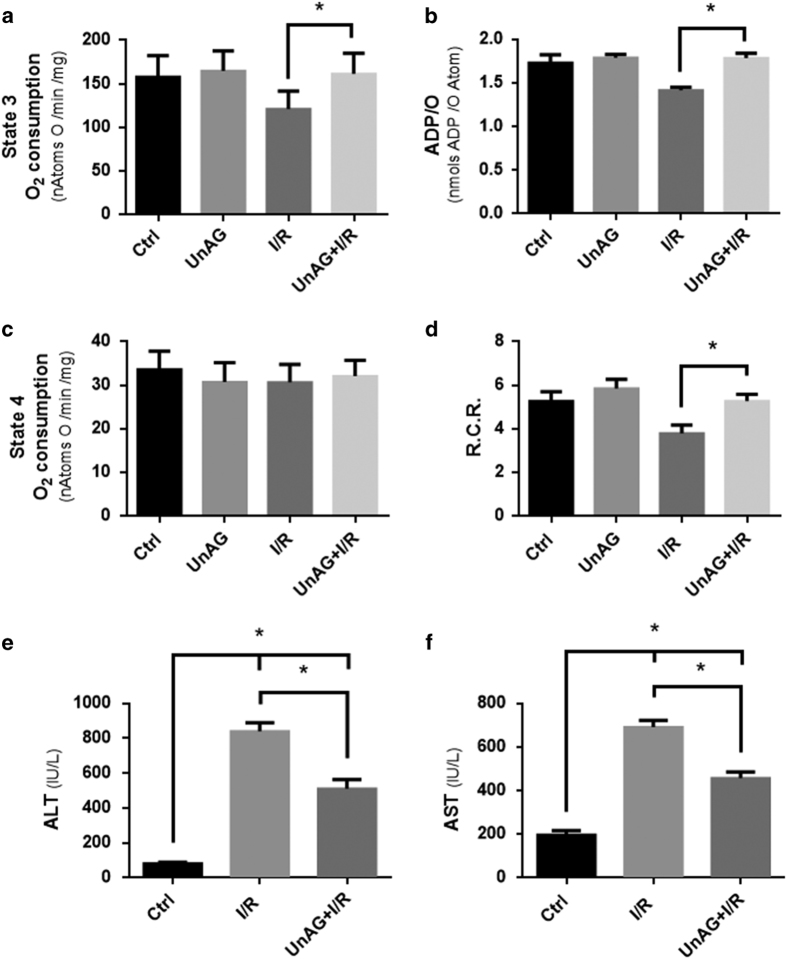
UnAG improves liver mitochondria respiration. (**a**) Histogram representation of oxygen consumption during State 3 (that is, ADP stimulated) respiration of liver mitochondria recovered from animals and treated as indicated (*P<0.05 UnAG+I/R *versus* I/R).Data are reported as mean±S.E.M. and representative of five different experiments, performed in triplicate (*N*=5). (**b**) Histogram representation of ADP/O ratio on liver mitochondria, treated as indicated; the absolute value reflects the nmoles of ADP phoshorylated per oxygen atom (*P<0.05 UnAG+I/R *versus* I/R). Data are reported as mean±S.E.M. and representative of five different experiments, performed in triplicate (*N*=5). (**c**) Histogram representation of oxygen consumption during post phosphorylative State 4 respiration on liver mitochondria, treated as indicated. Data are reported as mean±S.E.M. and representative of five different experiments, performed in triplicate (*N*=5). (**d**) Histogram representation of Respiratory Control Ratio (R.C.R.), the normalized index of ETC performance, which is obtained by dividing State 3 to State 4 (*P<0.05 UnAG+I/R *versus* I/R). Data are reported as mean±S.E.M. and representative of five different experiments, performed in triplicate (*N*=5). (**e**, **f**) Histogram representation of plasmatic concentration (International Units per liter) of the hepatic ALT (**e**) and AST (**f**) enzymes (the following both for ALT and AST: *P<0.05 UnAG+I/R *versus* I/R; *P<0.05 Ctrl *versus* UnAG+I/R and I/R). Data are reported as mean±S.E.M. and representative of five different experiments, performed in triplicate (*N*=5).

The oxygen consumption rate was evaluated in mitochondria isolated from liver subjected to I/R and treated with UnAG since mitochondria utilize oxygen to generate the membrane potential and eventually ATP. It was found that the oxygen consumption rate was negatively affected by ischemia during ADP-mediated State 3 respiration, while it was completely reverted by UnAG treatment (Figure 2a).

Data from mitochondrial respiration were further explored by means of the highly informative respiratory control (R.C.R.) and ADP/O ratio.^27^ It was observed that ADP/O ratio was increased by UnAG (Figure 2b). This observation, along with the finding that UnAG did not significantly change State 4 respiration (Figure 2c), suggests that the increase in oxygen consumption relies on ATP synthesis, rather than on an increase in proton leakage. In fact, R.C.R. increased upon UnAG treatment (Figure 2d). UnAG was therefore able to reduce I/R injury by improving mitochondrial bioenergetic efficiency. This is consistent with the normalization of transaminases level, alanine aminotransferase (ALT) and aspartate aminotransferase (AST), in UnAG-treated animals. As shown in Figures 2e and f, warm I/R significantly increased AST and ALT plasma levels relative to the control group. UnAG was effective in reducing the increase in the plasma levels of AST and ALT, as caused by I/R.

### UnAG improves specific ETC activity and increases CoxIV and Opa1 expression

**Figure 3:**
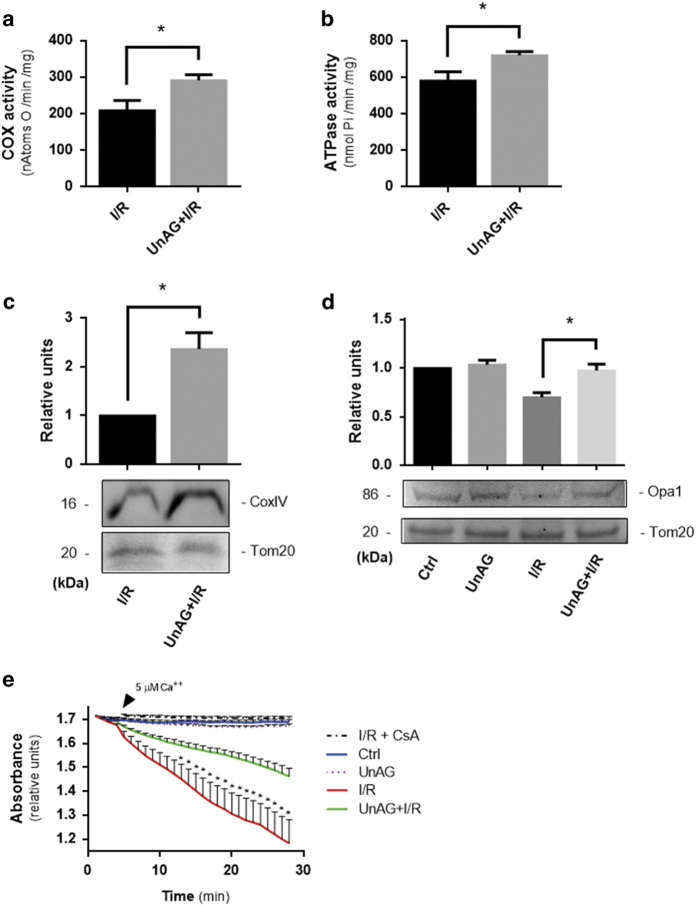
UnAG enhances specific ETC complex activities, induces CoxIV and Opa1 expression and promotes apoptosis resistance. (**a**) Histogram representation of Cytochrome *c* oxidase (COX) activity in mitochondria recovered from I/R animals treated as indicated (*P<0.05 UnAG+I/R *versus* I/R). Data are reported as mean±S.E.M. and representative of four different experiments, performed in triplicate (*N*=4). (**b**) Histogram representation of ATPsynthase activity in mitochondria, treated as indicated, expressed by reverse ATPase activity (*P<0.05 UnAG+I/R *versus* I/R). Data are reported as mean±S.E.M. and representative of five different experiments, performed in triplicate (*N*=5). (**c**) Mitochondria recovered from I/R animals, treated as indicated, were lysed and analyzed for CoxIV content, normalized to Tom20 (*P<0.05 UnAG+I/R *versus* I/R). Data are reported as mean±S.E.M. and representative of four different experiments, performed in triplicate (*N*=4). (**d**) Mitochondria recovered from animals, treated as indicated, were lysed and analyzed for Opa1 content, normalized to Tom20 (*P<0.05 UnAG+I/R *versus* I/R). Data are reported as mean±S.E.M. and representative of three different experiments, performed in triplicate (*N*=3). (**e**) Evaluation of mitochondrial permeability transition pore (mPTP) opening of liver mitochondria isolated from animals, treated as indicated. Calcium concentration (5 *μ*M) was added to individual preparations. I/R+Cyclosporine A (CsA) was used as a negative control for mPTP opening (*P<0.05 UnAG+I/R *versus* I/R from the 13th minute of assay). Data are reported as mean±S.E.M. and representative of five different experiments, performed in triplicate (*N*=5).

Specific ETC complex activities were assessed in mitochondria subjected to I/R damage in order to gain further insight into the mechanisms that account for UnAG action. It was found that UnAG improved COX functional activity, as shown by the oxygen consumption rate (Figure 3a). By contrast, no differences were detected when Complex II-succinate dehydrogenase-activity was analyzed (data not shown). This indicates the crucial role played by COX in sustaining the whole oxygen consumption rate (Figures 2a and d). In addition, it was proved that UnAG increased the reverse ATPase activity of mitochondrial ATPsynthase (Figure 3b).

The observation that COX activity was increased by UnAG treatment in an ischemic setting led us to investigate the levels of the nuclear-encoded subunit CoxIV in hepatic mitochondria recovered from untreated and treated-animals. As shown in Figure 3c, it was found that UnAG increased CoxIV expression but not that of CoxI and ATP synthase (data not shown).

Opa1 has recently emerged as an important regulator of the mitochondrial IM fusion process, as well as that of OXPHOS sensing and cristae remodeling.^9,10^ Opa1 content in mitochondria was therefore assessed as a means to further analyze the mechanisms of UnAG action. As shown in Figure 3d, ischemic liver mitochondria displayed increased levels of Opa1 when treated with UnAG.

### UnAG protects hepatocytes against intrinsic apoptosis

Mitochondria are also crucial for the apoptotic process. ATP deficiency leads to dysfunctional ATPase-mediated ion transport, which contributes to an increase in intracellular and mitochondrial calcium content. This, in turn, generates mitochondrial permeability transition pore (mPTP) opening and Cytocrome *c* release, resulting in the activation of the caspase cascade.^28–31^ Isolated mitochondria from animals subjected to differing experimental conditions were therefore additionally exposed to toxic calcium concentrations and mPTP was assessed, as mitochondrial swelling, in order to evaluate any possible UnAG contribution to protecting cells from the increase in calcium content and the activation of the apoptotic pathway. Cyclosporine A, which prevents mPTP opening, was used as a negative control. As shown in Figure 3e, delayed mPTP opening was found to occur in mitochondria recovered from livers not exposed to I/R. By contrast, toxic calcium concentration induced mPTP opening and osmotic swelling was found in mitochondria recovered from I/R animals. It is worth noting that calcium overload-mediated osmotic swelling was partially prevented in mitochondria from UnAG-treated animals. This suggests that UnAG may confer acquired resistance to mPTP opening and possibly apoptosis.

### UnAG effects are recapitulated *in vitro*

**Figure 4:**
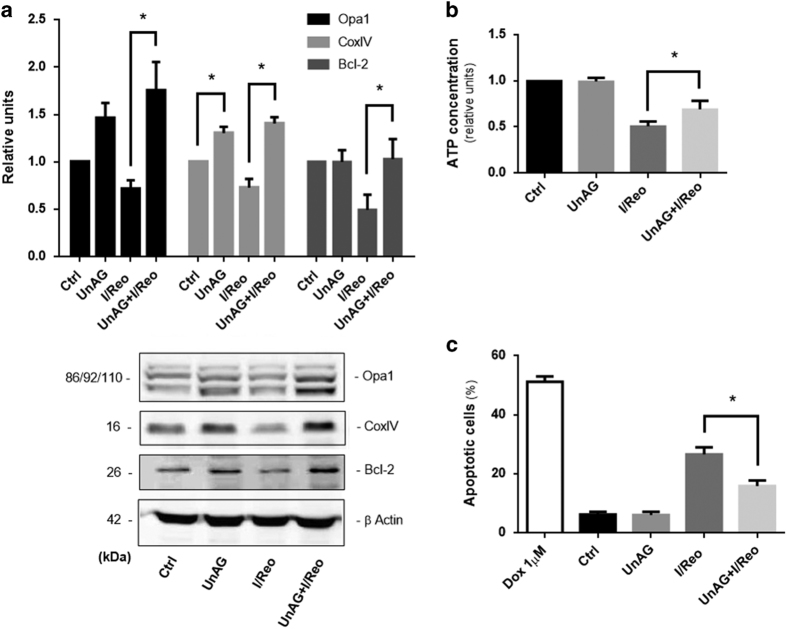
UnAG upregulates Opa1 expression resulting in ATP production and apoptosis resistance during *in vitro* ischemia/reoxygenation (I/Reo). (**a**) Cell extracts from RNT cells subjected to *in vitro* ischemia/reoxygenation (I/Reo), treated as indicated, were analyzed for Opa1, CoxIV and Bcl-2 content, normalized to *β* actin content (for Opa1 and Bcl-2, *P<0.05 UnAG+I/Reo *versus* I/Reo; for CoxIV, *P<0.05 UnAG *versus* Ctrl and *P<0.05 UnAG+I/Reo *versus* I/Reo). Data are reported as mean±S.E.M. and representative of three different experiments, performed in triplicate (*N*=3). (**b**) Histogram representation of the relative cellular ATP content. Data are obtained from RNT cells, treated as indicated (*P<0.05 UnAG+I/Reo *versus* I/Reo). Data are reported as mean±S.E.M. and representative of four different experiments, performed in triplicate (*N*=4). (**c**) Apoptosis assay was performed on RNT cells subjected to I/Reo treated as indicated. Doxorubicin (1 *μ*M) was used as a positive control (*P<0.05 UnAG+I/Reo *versus* I/Reo). Data are expressed as percentage±S.E.M. of total apoptotic cells, and representative of three different experiments, performed in triplicate (*N*=3).

UnAG effects were investigated in an *in vitro* model of I/R damage in order to validate the above *ex vivo* results. For this purpose, cultured immortalized hepatocytes were serum deprived and subjected to 24 h of *in vitro* ischemia followed by 3 h of oxygen reperfusion (I/Reo) and then either treated with UnAG or left untreated. UnAG upregulated CoxIV and Opa1 expression (Figure 4a), as is consistent with the *ex vivo* data. This translated into increased cellular ATP content (Figure 4b). Moreover, when the expression of Bcl-2 and the number of apoptotic cells were analyzed (Figures 4a and c), it was found that UnAG was able to protect liver cells from the activation of the intrinsic apoptotic pathway triggered by I/Reo, even *in vitro*.

### Opa1 is crucial for UnAG-mediated protection against I/Reo injury

**Figure 5:**
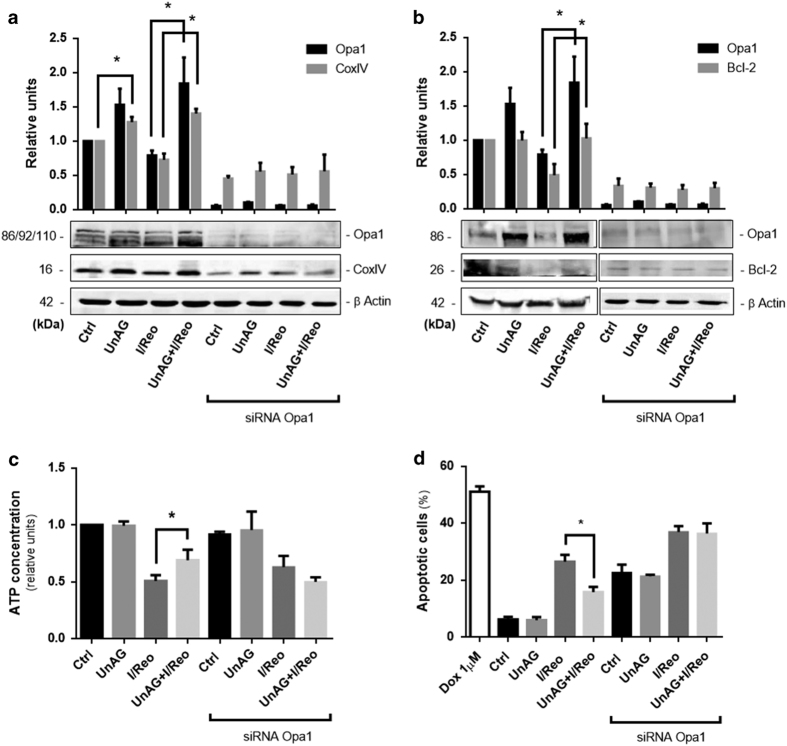
Opa1 is required for UnAG-mediated protection against I/R injury. (**a**, **b**) Cell extracts from either Opa1-silenced or control RNT cells, treated as indicated, were analyzed for Opa1,CoxIV and Bcl-2 content, normalized to *β* actin content (for Opa1 and Bcl-2, *P<0.05 UnAG+I/Reo *versus* I/Reo; for Cox IV, *P<0.05 UnAG *versus* Ctrl and*P<0.05UnAG+I/Reo *versus* I/Reo). Data are reported as mean±S.E.M. and representative of four different experiments, performed in triplicate (*N*=4). Figure **b** has been cropped. (**c**) Histogram representation of the relative cellular ATP content. Data are obtained from cells, treated as indicated (*P<0.05 UnAG+I/Reo *versus* I/Reo). Data are reported as mean±S.E.M. and representative of four different experiments, performed in triplicate (*N*=4). (**d**) Apoptosis assay was performed on RNT cells subjected to I/Reo, treated as indicated. Doxorubicin (1 *μ*M) was used as a positive control (*P<0.05 UnAG+I/Reo *versus* I/Reo). Data are reported as percentage±S.E.M. of total apoptotic cells, and representative of four different experiments, performed in triplicate (*N*=4).

**Figure 6:**
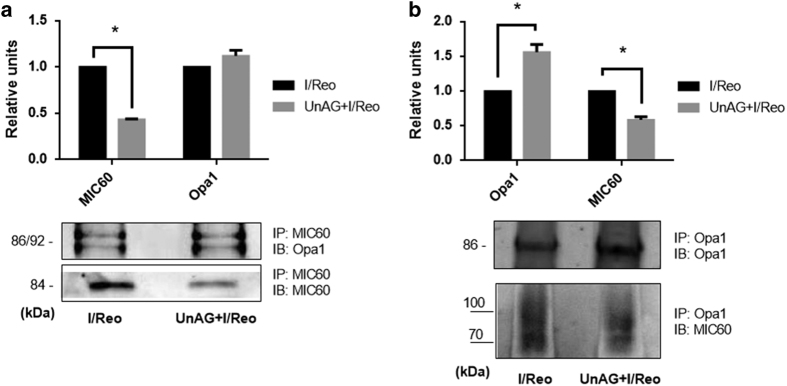
UnAG impacts on Opa1-MIC60 interaction. (**a**) Representative co-immunoprecipitation (co-IP) of MIC60 from cell extracts (1 mg) of RNT cells treated as indicated, blotted with Opa1 and normalized to MIC60 (for MIC60, *P<0.05 UnAG+I/Reo *versus* I/Reo). (**b**) Representative co-IP of Opa1 from cell extracts (1 mg) of RNT cells, treated as indicated, blotted with MIC60 and normalized to Opa1 (*P<0.05 UnAG+I/Reo *versus* I/Reo, for MIC60 and Opa1). Data are reported as mean±S.E.M. and representative of four different experiments, performed in triplicate (*N*=4).

The role of Opa1 in maintaining mitochondrial cristae structure to avoid ATP production loss has been reported.^32^ siRNA technology was used on cells subjected to I/Reo and UnAG treatment in order to investigate the biological relevance of Opa1 in mediating UnAG action on mitochondria. The results reported in Figure 5 clearly demonstrate that UnAG action strictly depends on Opa1 expression in I/Reo conditions. Indeed, it was found that the increase of both CoxIV and Bcl-2 expression was lost in cells depleted of Opa1 and treated with UnAG (Figures 5a and b). As a matter of fact, UnAG failed to increase cellular ATP content and resistance to apoptosis in Opa1-depleted cells (Figures 5c and d). It has been previously demonstrated that the interplay between Opa1 and MIC60 is crucial for mitochondrial remodeling.^33^ We therefore investigated whether this interplay could occur also in our model. As shown in Figures 6a and b co-immunoprecipitation experiments demonstrated that UnAG could also be able to regulate cristae shape, in ischemic conditions, by increasing Opa1 content in the Opa1/MIC60 complex.

## Discussion

Liver I/R injury increases morbidity and mortality in clinical settings, including liver transplantation, hepatic surgery and hepatic failure after hemorrhagic shock.^3,34^ Mechanistically, liver I/R injury is accompanied by impaired mitochondrial structure and function, which leads to a decline in mitochondrial respiration and enhanced ROS production.^35,36^ A number of therapeutic options have been proposed, but have failed to improve long-term outcomes.^20,21,34^ This failure to obtain a clinical response from current therapeutic options has driven the development of alternative strategies that are mainly focused on improving antioxidant machinery. We have previously shown that UnAG, the more abundant circulating form of ghrelin, exerts anti-oxidant effects on numerous cell types,^23–25^ and a proof of concept for its role in improving mitochondrial dysfunction has been reported in preclinical settings of ischemic injury.^24,25^ In accordance with our previous data, we have shown herein that UnAG improves mitochondrial function in a rat model of hepatic I/R injury. Higher oxygen consumption ensures better ATP availability, particularly when there is a special need for energetic substrates to keep vital cellular processes and escape apoptosis and necrosis. Furthermore, an efficient respiratory control index, ADP/O and state 3 respiration are crucial for mitochondria homeostasis.^27^ Our results consistently demonstrate that I/R-induced ETC-metabolic impairment and oxygen consumption rate are rescued by UnAG administration. In line with this observation, it was found that R.C.R. and the ADP/O ratio were increased in mitochondria recovered from UnAG-treated animals. This suggests that UnAG may reduce I/R injury by improving mitochondrial metabolic functions. Mitochondria possess their own genome that encodes ribosomal RNAs, transfer RNAs and proteins.^37^ These polypeptides are the essential catalytic components of ETC complexes I, III and IV and ATP synthase which form the OXPHOS.^37^ As expected, dysfunctional mitochondria showed impaired oxidative phosphorylation and ATP generation in hepatic I/R injury. By contrast, it was found that *in vivo* UnAG treatment led to an improvement in ATP synthase activity and to the increased expression of CoxIV, which is mechanistically consistent with the enhancement of mitochondrial performance.

A lack of oxygen interferes with oxidative phosphorylation and hampers ATP production, which, in turn, causes unbalanced cytoplasmic ion levels, mainly involving Ca^2+^.^38^ Such derangement translates into the activation of the intrinsic apoptosis pathway via the mPTP opening. mPTP is a dynamic structure closed in the ischemic period, while opened during reperfusion. mPTP opening leads to membrane potential dissipation, uncoupled oxidative phosphorylation, resulting in ATP depletion, and the release of apoptotic drivers, such as Cytochrome *c.*^5,35,39^ The protection of mitochondrial function exerted by UnAG during liver I/R injury is also sustained by the inhibition of mPTP opening, a reduction in the number of apoptotic cells and increased Bcl-2 expression. As a matter of fact, cellular ATP content is also increased upon UnAG treatment. The suppression of glycogen synthase kinase 3*β* has been previously reported as a mechanism that regulates hepatic mPTP opening in I/R injury.^40^ Although we did not gain further insight into the molecular mechanism, our data indicate that UnAG protection against I/R injury in the liver may be the result of multiple mechanisms involving both the improvement of mitochondrial respiratory capacity and apoptosis resistance.

Mitochondria dynamically regulate their organization via opposing fusion and fission processes to maintain bioenergetic homeostasis and contribute to key cellular paths.^8^ Mitochondria undergo fission to generate fragmented organelles required for cell division and mitophagy, while they go through fusion to form elongated interconnected networks which allow the renewal of damaged mitochondrial DNA to occur.^41^ Although mitochondrial fusion is orchestrated by different mitochondrial proteins, including Mfn1, Mfn2 and Opa1, data from genetic ablation demonstrated that, unlike Opa1 deficiency which led to mitochondrial dysfunction,^32^ Mfn1 and Mfn2 rescued a deficiency in one or the other at least in part. Genetic data are mechanistically consistent with the role of Opa1 in maintaining cristae structure within mitochondria, in preserving IM integrity and IM potential and in avoiding the release of Cytochrome *c* from cristae.^32^ Therefore, increased content of Opa1 upon UnAG challenge might play a key role for the enhanced mitochondrial performance observed in our experimental model.

Opa1 has also been reported to promote CJ formation.^9,42,43^ Besides Opa1, MICOS has also recently been reported to play a role in CJ biogenesis.^17^ As previously described in other tissue,^17^ it was found herein that Opa1 and MIC60 physically interact and that the amount of Opa1 in the molecular complex formed is increased in response to UnAG in the I/R setting. Increased Opa1 in skeletal and cardiac muscle, the brain and liver, confer remarkable protection against a widespread spectrum of tissue injury.^44^ Moreover, Civiletto *et al.*^45^ have demonstrated that increased Opa1 content can also ameliorate defective mitochondrial bioenergetics. Furthermore, Glytsou *et al.*^17^ have reported that Opa1 epistatically influences core MIC60 in CJ biogenesis and identified Opa1 as a key regulator of apoptotic cristae remodeling. The MIC60/OPA1 complex is essential for mitochondrial CJ formation and remodeling. OPA1 functionally interacts with MIC60, but such a complex is not necessary for CJ formation.^17^ Mitochondria are swollen and mitochondrial structure and function are compromised after I/R, leading to a need for remodeling.^46^ The protection afforded by the hormone, with the previous administration of UnAG, prevents structural and functional damage to the mitochondria population, which is why there is a minor need for crosstalk between the cristae regulators, OPA1/MIC60. This is supported by the work of Barrera *et al.*,^33^ in which OPA−/− animals display an increase in MIC60 content.

**Figure 7:**
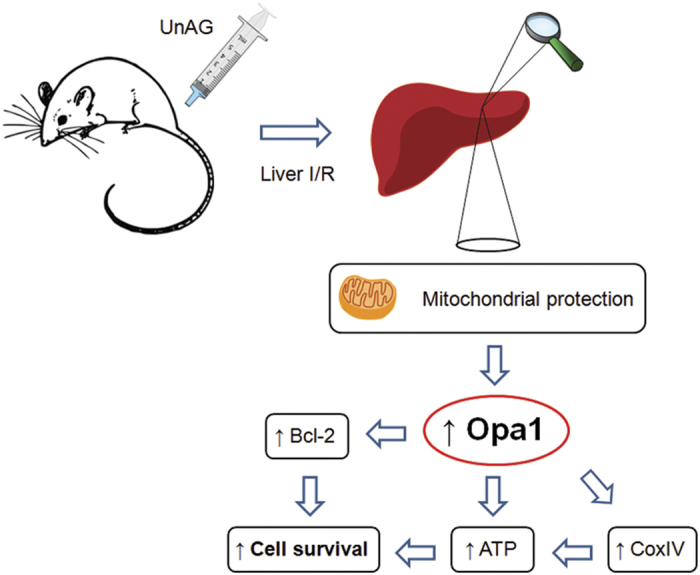
Schematic representation of UnAG action on liver mitochondria subjected to I/R. Following liver mitochondria I/R injury, UnAG rescues oxygen consumption rate and improves ATP synthase activity. Its effects are mediated by the increased expression of Opa1. The upregulation of Opa1 leads to increased CoxIV expression and subsequently improved ATP content, which, together with Bcl-2 upregulation, translates into enhanced cell survival. UnAG, Unacylated Ghrelin; Opa1, Optic atrophy 1; Bcl-2, B-cell lymphoma-2; CoxIV, Cytochrome oxydase subunit four.

In conclusion, the present study provides the first evidence that UnAG may limit liver damage during I/R injury, upon acting on the mitochondrial level where it is able to protect respiratory chain function and to limit both ATP depletion and proton leak (Figure 7). Prompt mitochondrial biogenesis has already been suggested as a strategy for the clinical management of liver I/R injury,^47^ and, in fact, our data suggest that UnAG may be exploited as a new therapeutic option for the prevention of liver damage and/or the targeting of primary mitochondrial disorders by ameliorating mitochondrial bioenergetics.

## Materials and methods

### Reagents and antibodies


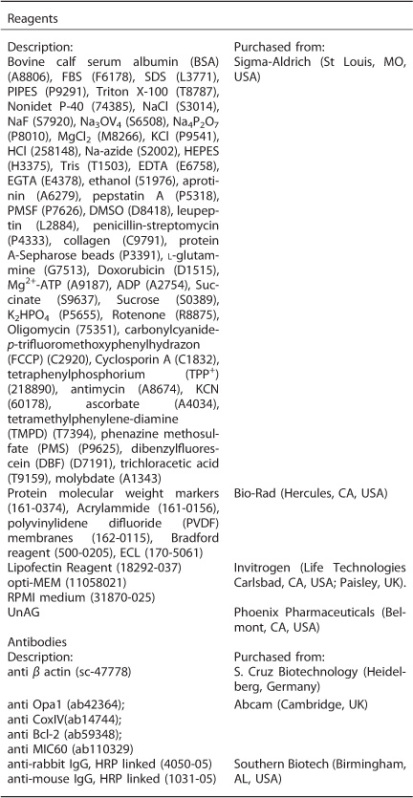


### Hepatic I/R rat model

Ten 16-week–old Wistar rats (Charles River, France) were maintained on a standard laboratory diet. The animals were given food and water *ad libitum* with a 12 h light/dark daily cycle and were acclimated for 1 week before the start of the experiment. Animal studies were conducted in accordance with the Portuguese National Institute of Health Guide for the Care and Use of Laboratory Animals. All procedures were approved by the Ethics Committee of the University of Coimbra. Rats were housed according with the Federation of European Laboratory Animal Science Association Guidelines. All experiments were performed in accordance with relevant guidelines and regulations. Rats were randomly allocated to receive either hepatic I/R or I/R plus UnAG (100 *μ*g/kg, injected into hepatic artery 15 min before ischemia).

Animals were anesthetized with ketamine (50 mg/kg) and chlorpromazine (50 mg/kg). A model of partial ischemia (70%) was used to prevent mesenteric venous congestion by permitting portal decompression through the right and caudate lobes. After a midline laparotomy, the hepatic artery and portal vein to the left and median liver lobes were occluded for 90 min. Reperfusion (2 h) was initiated by removal of the clamp.^48^

### Isolation of liver mitochondria

Animals were killed by cervical dislocation immediately after 2 h reperfusion. Mitochondria were isolated via differential centrifugation, as previously described^49^ and kept on ice all throughout the procedure. The homogenization medium contained 250 mM sucrose, 10 mM HEPES (pH 7.4), 0.5 mM EGTA and 0.1% fat-free bovine serum albumin. EGTA and bovine serum albumin were omitted from the final washing medium, which was adjusted to pH 7.4. Briefly, after the homogenization of the minced blood-free hepatic tissue, the homogenate was centrifuged at 500×*g* for 10 min at 4 °C. The resulting supernatant was spun at 10 000×*g* for 10 min at 4 °C to pellet the mitochondria, which were resuspended in a final washing medium. Protein content was determined using the Biuret method,^50^ calibrated with bovine serum albumin.

### Mitochondrial respiration

Oxygen consumption was polarographically determined using a Clark type polarographic oxygen sensor, as previously described.^49^ Mitochondria (1 mg) were suspended under constant stirring at 25 °C in 1 ml of standard respiratory medium (130 mM sucrose, 50 mM KCl, 5 mM MgCl_2_, 5 mM KH_2_PO_4_, 50 *μ*M EDTA, and 5 mM HEPES (pH 7.4)) and energized by adding glutamate/malate or succinate to a final concentration of 5 mM. Rotenone (2 *μ*M), an inhibitor of complex I, was added earlier to the succinate assays. State 3 respiration was induced by adding 200 nmoles ADP. Oxygen consumption was also measured in the presence of oligomycin (0.5 *μ*g/mg protein) and 1 *μ*M carbonylcyanide-*p*-trifluoromethoxyphenylhydrazon (FCCP). States 3 and 4 and respiratory control ratio (R.C.R.) were calculated according to Chance and Williams.^51^ All experiments were performed in triplicate.

### Mitochondrial transmembrane potential (Δ*ψ*) measurements

Mitochondrial transmembrane potential (Δ*ψ*) was estimated using an ion-selective electrode to measure the distribution of tetraphenylphosphorium (TPP^+^) according to previously established methods.^52,53^ The voltage response of the TPP^+^ electrode to log [TPP^+^] was linear with a slope of 59±1, in conformity with the Nernst equation. Reactions were carried out at 25 °C, in a temperature-controlled water-jacketed chamber with magnetic stirring. Mitochondria (1 mg) were suspended in 1 ml of standard respiratory medium (as in mitochondrial respiration), supplemented with 3 *μ*M TPP^+^. A matrix volume of 1.1 *μ*l/mg protein was assumed. All experiments were performed in triplicate.

### Enzymatic activities

Succinate dehydrogenase activity was polarographically determined as previously described.^54^ The reaction was carried out at 25 °C in 1.4 ml of standard respiratory medium (as in mitochondrial respiration) supplemented with 5 mM succinate, 2 *μ*M rotenone, 0.1 *μ*g Antimycin A, 1 mM KCN and 0.3 mg Triton X-100. After the addition of freeze-thawed mitochondria (0.25 mg) the reaction was initiated with 1 mM phenazine methosulfate.

Cytochrome *c* Oxidase activity was polarographically determined, as previously described.^55^ The reaction was carried out at 25 °C in 1.4 ml of standard respiratory medium supplemented with 2 *μ*M rotenone, 10 *μ*M oxidized Cytochrome *c*, 0.3 mg Triton X-100 and freeze-thawed mitochondria (0.25 mg), after which the reaction was initiated by adding 5 mM ascorbate plus 0.25 mM tetramethylphenylene-diamine.

ATPase activity was determined spectrophotometrically at 660 nm, in association with ATP hydrolysis.^56^ The reaction was carried out at 37 °C, in 2 ml reaction medium (100 mM NaCl, 25 mM KCl, 5 mM MgCl2 and 50 mM HEPES, pH7.4). After the addition of freeze-thawed mitochondria (0.25 mg), dibenzylfluorescein was added and allowed to incubate for 3 min before the initiation of the reaction with the addition of 2 mM Mg^2+^-ATP, in both the presence or absence of oligomycin (1 *μ*g/mg protein). The reaction was stopped after 10 min by adding 1 ml of 40% trichloroacetic acid and the samples were centrifuged (5 min, 3000×*g*). 2 ml of ammoniummolybdate (1%) plus 2 ml H_2_O were then added to 1 ml of supernatant and reacted for 5 min at room temperature. ATPase activity was calculated as the difference in total absorbance and the absorbance in the presence of oligomycin. All experiments were performed in triplicate.

### Measurement of the mitochondria permeability transition

Mitochondrial swelling was estimated by changes in light scattering, as spectrophotometrically monitored at 540 nm.^57^ Reactions were carried out at 25 °C. Experiments were started by the addition of mitochondria (1 mg) to 2 ml of reaction medium (200 mM sucrose, 10 mM Tris-MOPS, 1 mM KH_2_PO_4_, 10 *μ*M EGTA, pH 7.4) supplemented with 3 *μ*M rotenone, 0.5 *μ*g oligomycin,5 mM succinate and, in selected assays, Cyclosporin A 1 *μ*M, a known mPTP inhibitor as a negative control. After a brief period for the recording of basal absorbance, toxic amount of Ca^2+^ (5 *μ*M) was added and the resulting alterations in light scattering were registered, as previously described.^57^ All experiments were performed in triplicate.

### Cell culture and *in vitro* I/Reo assay

A long-term stable hepatocyte cell line (RNT)^58^ was cultured in RPMI 1640 complete medium with 10% FBS (Lonza Group Ltd, Basel, Switzerland) in a 5% CO_2_ atmosphere at high density in collagen-coated flasks grown to subconfluence prior to experiments. RNT cells were cultured for 24 h in RPMI 1640 with 5% FBS either alone or in combination with UnAG (1 *μ*M) as indicated.^24,25^ On day 2, cells were subjected to *in vitro* ischemia, which was induced by incubating cells in RPMI 1640 plus 2% FBS in a 5% CO_2_, 95% N_2_ humidified atmosphere, yielding 1% O_2_ concentrations for 24 h.^59^ Subsequently, cells were reoxygenated with 75% N_2_, 20% O_2_and 5% CO_2_ for 3 h.^59^

### ATP content assay

ATP content was performed using an ATP assay kit, according to the manufacturer’s instructions (Sigma-Aldrich), to determine the level of cellular ATP content as an indirect measurement of viable cells. Briefly, RNT cells, that were treated as indicated, were subjected to *in vitro* ischemia for 24 h as described above. After reoxygenation, ATP was quantitatively determined by measuring luminescence generated in an ATP-dependent luciferin-luciferase bioluminescence assay. A standard curve was used in each experiment and the samples were diluted to be in the linear range of the standard curve. All experiments were performed in triplicate.

### Silencing of the endogenous Opa1 by small interfering RNAs (siRNAs)

RNT cells were transiently transfected with siRNA for Opa1, or with duplex siRNAs (Qiagen, Valencia, CA, USA),^25^ and treated as indicated in order to obtain Opa1 inactivation. Transfection was performed according to the manufacturer’s instructions. Whole cell extracts were processed 48 h after transfection. Cell viability was evaluated at the end of each experiment.

### Western blot analysis

Cells were lysed (50 mM Tris-HCl (pH 8.3), 1% Triton X-100, 10 mM PMSF, 100 U/ml aprotinin, 10 *μ*M leupeptin) and protein concentrations were obtained, as previously described. Proteins (50 *μ*g) were subjected to SDS-PAGE, transferred onto nitrocellulose membranes, blotted with the indicated antibodies and revealed using an enhanced chemiluminescence detection system.^25^ Densitometric analysis was used to calculate the differences in the fold induction of protein levels and normalized to *β*-actin. Values are reported as relative amounts and all experiments were performed in triplicate.

### Immunoprecipitation

Protein concentration was determined using the Bradford dye-binding procedure. Equal amounts of cell extracts (1 mg) were immunoprecipitated with the indicated antibody and immunocomplexes were bound to protein-A-Sepharose beads and recovered by centrifugation.^60^ Bound material was eluted by boiling beads in 1% SDS, then separated on 8% SDS-PAGE in reducing conditions and processed as described in the western blot section. All experiments were performed in triplicate.

### Apoptosis assay

For apoptosis assay, RNT cells, cultured in different experimental conditions, were subjected to Muse Annexin V and cell dead assay (Merck, Darmstadt, Germany) in accordance with the manufacturer’s instructions. All experiments were performed in triplicate.

### ALT and AST level determination

Plasma samples were collected immediately after the killing and enzymatic determinations of ALT and AST were performed using commercial kits (Hospitex Diagnostics). All experiments were performed in triplicate.

### Statistical analysis

Data are represented as a mean of *n*⩾3±S.E.M., while statistical significance was determined using the one-way (two-way for the swelling analysis) ANOVA test with a Bonferroni correction (GraphPad Prism software, La Jolla, CA, USA). A value of *p*<0.05 (*) was considered statistically significant. According to our previous data, the minimum sample size that will permit us to detect a 30% difference between the experimental groups, with 90% power and a probability level of 0.05 in a two-tailed hypothesis, was *n*=5 rats/group.

## Additional information

**Publisher’s note:** Springer Nature remains neutral with regard to jurisdictional claims in published maps and institutional affiliations.
